# IL-10Rα expression is post-transcriptionally regulated by miR-15a, miR-185, and miR-211 in melanoma

**DOI:** 10.1186/s12920-015-0156-3

**Published:** 2015-12-03

**Authors:** Isabella Venza, Maria Visalli, Concetta Beninati, Salvatore Benfatto, Diana Teti, Mario Venza

**Affiliations:** Department of Clinical and Experimental Medicine, Azienda Policlinico Universitario G. Martino, viaConsolare Valeria, 1, Messina, 98125 Italy; Department of Human Pathology of Adult and Developmental Age “Gaetano Barresi”, Azienda PoliclinicoUniversitario G. Martino, via Consolare Valeria, 1, Messina, 98125 Italy; Scylla Biotech Srl, University of Messina, Messina, Italy

**Keywords:** IL-10, IL-10Rα, IL-10Rβ, miRNAs, Cutaneous melanoma, Uveal melanoma

## Abstract

**Background:**

IL-10 is an immunoregulatory cytokine that increases during malignant diseases. The purpose of this study was to: i) determine the mRNA amounts of *IL-10*, *IL-10Rα*, and *IL-10Rβ* in cutaneous and uveal melanoma cells and specimens; ii) evaluate their post-transcriptional regulation by miRNAs; iii) ascertain whether miRNA dysregulation may affect IL-10-induced proliferation.

**Methods:**

Genome-wide miRNA expression profiling was performed using a human microarray platform. The reference gene mRNA was measured through qPCR. miRNAs/mRNAs interactions were predicted by TargetScan, microRNA, and PITA. Transfections of specific miRNA mimics/inhibitors were carried out. Cell proliferation was assessed by MTT assay in the presence of IL-10 after transfection with miRNA mimics/inhibitors.

**Results:**

There were no differences in IL*-*10 mRNA levels between any of the 3 melanoma cell lines tested and normal melanocytes. However, lower *IL-10Rα* expression was found in G361 and OCM-1 cells, and higher levels of *IL-10Rβ* were observed in G361 cells compared with normal melanocytes. GR-M cells did not exhibit any modifications in *IL-10Rα* and *IL-10Rβ* expression. miR-15a, miR-185, miR-211, and miR-30d were upregulated in G361 and OCM-1 cells, remaining at similar levels in GR-M cells. miR-409-3p and miR-605were down-regulated exclusively in G361 cells. Prediction tools revealed that miR-15a, miR-185, and miR-211 targeted *IL-10Rα* whereas none of the miRNAs exclusively downregulated in G361 cells targeted *IL-10Rβ*. Luciferase reporter and western blot assays showed that *IL-10Rα* expression is directly regulated by miR-15a, miR-185, and miR-211, either alone or in combination. An inverse expression pattern between *IL-10Rα*, on one side, and miR-15a, miR-185, and miR-211 on the other one was also shown in melanoma samples. Ectopic expression of individual miR-15a, miR-185, and miR-211, and even more their co-expression, caused a marked decrease in the proliferation rate of all the cell lines. Likewise, inhibition of any specific miRNA promoted cell growth, an effect that further increased when inhibition concerned all three miRNA. Moreover, specific knockdown of IL-10Rα prevented the proliferative effect of miRNA inhibitors.

**Conclusions:**

Our results support a key role of *IL-10Rα* in the development and progression of melanoma and suggest that the IL-10/IL-10 receptor system may become a new therapeutic target for melanoma treatment.

## Background

IL-10 is generally believed to repress the inflammatory response [1] and immune reactions against a variety of tumors [2, 3]. This cytokine is secreted by immune and non-immune cells [4] and its production was found to increase during malignant diseases, including melanoma [5].

The ability of IL-10 to affect melanoma growth under a variety of conditions renders this cytokine an interesting topic of research in this field, although the mechanisms involved are complex and incompletely understood [6–8]. Some studies suggested that high local levels of this cytokine may favor melanoma growth by suppressing the activities of tumor-infiltrating cells involved in anti-tumor immunity. For example, it was shown that IL-10 can decrease proinflammatory cytokine expression [9] or anti-tumor T cell responses [7, 9]. These data suggested that the production of IL-10 by melanoma cells and its release in the surrounding microenvironment might produce a paralysis of the anti-melanoma immune response [5].

In contrast, other studies indicate that IL-10 may augment the effects of anti-melanoma vaccination [10] or inhibit melanoma metastasis through activation of NK cells [6]. Moreover, it was reported that IL-10 repressed tumor growth and the metastatic potential of melanoma cells by inhibiting the expression of angiogenic factors and, thereby, vascularization [8]. Irrespectively of the effects of IL-10 on the immune response, the direct effects of this cytokine on melanoma cell themselves have been only incompletely addressed, although one study suggested that IL-10 might function as an autocrine growth factor [7].

Expression of the IL-10Ron the cell surface is absolutely required for the responsiveness to this cytokine, but its role is in the context of melanoma development is ill-understood. IL-10R is composed of two different chains (IL-10Rα and IL-10Rβ). The alpha chain binds directly to IL-10 and the beta chain is subsequently recruited into the IL-10/IL-10Rα complex. Although it is generally believed that signal transduction pathways are triggered only after binding of the beta chain, it cannot be ruled out that this occurs also during initial binding of IL-10 to IL-10Rα [4].

Emerging evidence suggests a role for epigenetic modulation in melanomagenesis. In particular, miRNAs have been recognized as important mediators of biological processes linked to melanoma, including growth, cell cycle, invasiveness, migration, and immune evasion [11]. Given that very few data are available about the post-transcriptional regulation of the IL-10 system and its role in melanoma cell behavior, we aimed at investigating the involvement of miRNAs in the expression levels of IL-10 and its receptors in cutaneous and uveal melanoma cells.

## Methods

### Cell cultures

G361 cutaneous melanoma cells (ECACC, European Collection of Cell Cultures, Salisbury, UK) were grown in McCoy’s 5a medium modified with 10 % FBS, 2 mM L-glutamine, and 1 % penicillin/streptomycin. The cutaneous melanoma cell line GR-M (ECACC, European Collection of Cell Cultures, Salisbury, UK) and the uveal melanoma cell line OCM-1 (provided by J. Mellon, Department of Ophthalmology, UT Southwestern Medical Center, Dallas, TX) were cultured in RPMI 1640 medium supplemented with 2 mM L-glutamine, 1 % penicillin/streptomycin, and 10 % FBS.

### Tumor specimens

To exclude that our observations were restricted to cell lines maintained in long-term culture, we also examined primary tumors. Formalin-fixed paraffin-embedded (FFPE) tissue sections of 52 cutaneous melanomas (31 females and 21 males, age ranging from 45 to 65 years), 41 uveal melanomas (15 females and 26 males, age ranging from 52 to 68 years), and 35 normal skin specimens, taken from patients of the University Hospital of Messina, were examined. The investigation adhered to the Declaration of Helsinki and was approved by the Ethics Committee of the University Hospital of Messina. An informed verbal consent was given by patients.

### Total RNA extraction, reverse transcription and qPCR

Total RNA was extracted by TRIzol Reagent (Invitrogen). RNA samples were quantified with a Nanodrop 1000 spectrophotometer and their quality was evaluated using Agilent RNA 6000 Nano Assay. RNA samples were converted into cDNA using IMProm-II™ reverse transcriptase kit (Promega). Quantitative Real-Time PCR was performed by ABI Prism 7500 Real-Time PCR System (Applied Biosystems, Milan, Italy) with SYBR Green Mastermix (Applied Biosystems, UK). Primers were previously reported [12, 13]. The mRNA levels of *IL-10*, *IL-10Rα*, and *IL-10Rβ* were normalized to endogenous *β-*actin (Applied Biosystems).

### miRNA microarray

miRNA microarray experiments were performed using a human miRNA microarray platform (Agilent Sanger miRBase, release 10.1) with 723 human and 76 human viral miRNAs represented. Agilent Feature Extraction Software was used for background subtraction. LOWESS and Quantile normalizations were performed.

### Oligonucleotide transfection

MiR-15a, miR-185, and miR-211 mimics/inhibitors (Qiagen, Milan, Italy) were transfected either alone or in combination into melanoma cells using HiPerFect according to the manufacturer’s protocols (Qiagen). Cells were transfected twice with 100 pmol of oligonucleotide per well (0.5 × 10^6^ cells) at 24 h intervals. Transfected cells were assayed 48 h after the second transfection.

### Western blot analysis

Total cell extracts (50 μg) were resolved by SDS-PAGE and blotted onto nitrocellulose membranes with specific antibodies against IL-10 (sc-8438, Santa Cruz Biotechnology), IL-10Rα (sc-984, Santa Cruz Biotechnology), and IL-10Rβ (sc-514822, Santa Cruz Biotechnology).

### Plasmids constructs and transient transfections

The 3′-UTR of IL-10Rα and a mutation sequence were amplified by PCR using the primers with a Bgl II restriction site on each 5′ or 3′ strand. The PCR products were inserted into the Bgl II sites of the pGL3-control vector (Promega, Madison, WI, USA) and identified by DNA sequencing. The wild-type plasmids were created containing the 3′-UTR of IL-10Rα with complementary sequence of miR-15α ( IL-10Rα 3′-UTR wild 1), miR-185 (IL-10Rα 3′-UTR wild 2), miR-211 ( IL-10Rα 3′-UTR wild 3) or covering all the three miRNA side sites (IL-10Rα 3′-UTR wild-full-length). Four mutant plasmids were generated with the mutation sequence without complementary sequence of miR-15a (pGL3- IGF-1 3′-UTR mut 1), miR-185 (pGL3- IGF-1 3′-UTR mut 2), and miR-211 (pGL3- IGF-1 3′-UTR mut 3) or all of them (IL-10Rα 3′-UTR mut-full-length). For the luciferase reporter assay, cells were seeded on 24-well plates and co-transfected using Lipofectamine 2000 (Invitrogen) with 100 ng per well of the resulting luciferase UTR-report vector, 2 ng per well of pRLCMV vector (internal control, Promega), and 20 ng per well of miR-15a, miR-185, and miR-211 mimics or inhibitors following the manufacturer’s instructions (Qiagen, Milan, Italy). After 24 h, the cells were lysed, and the relative luciferase activity was assessed with the Dual-Luciferase Assay Reporter System (Promega). For transient knockdown of *IL-10Rα*, cells were transfected with specific small interfering RNA (siRNA) targeting *IL-10Rα* or non-targeting control siRNA (Santa Cruz Biotechnology, Milan, Italy) 24 h after plating using Lipofectamine 2000 (Invitrogen) with the siRNA at a final concentration of 100 nM.

### Cell proliferation assay

Cells were treated with recombinant human IL-10 (R & D Systems) at various doses (50, 100, or 500 U/ml) and for different times (at 6-hr intervals during a 72-hr culture period) accordingly to previously reported conditions [7]. Proliferation was measured using the MTT Assay Kit (Cayman Chemical Company, Michigan, USA).

### Densitometry and statistical analysis

The one-way analysis of variance (ANOVA) test, followed by a pair-wise multiple comparison test (Bonferroni t test), was performed to identify the differences among the groups. The relative intensities of protein bands were analyzed by Image J software (Bethesda, MD, USA). Statistical significance was assigned when the p value was <0.05.

## Results

### Expression levels of *IL-10*, *IL-10Rα*, and *IL-10Rβ* in cutaneous and uveal melanoma cells

**Fig. 1:**
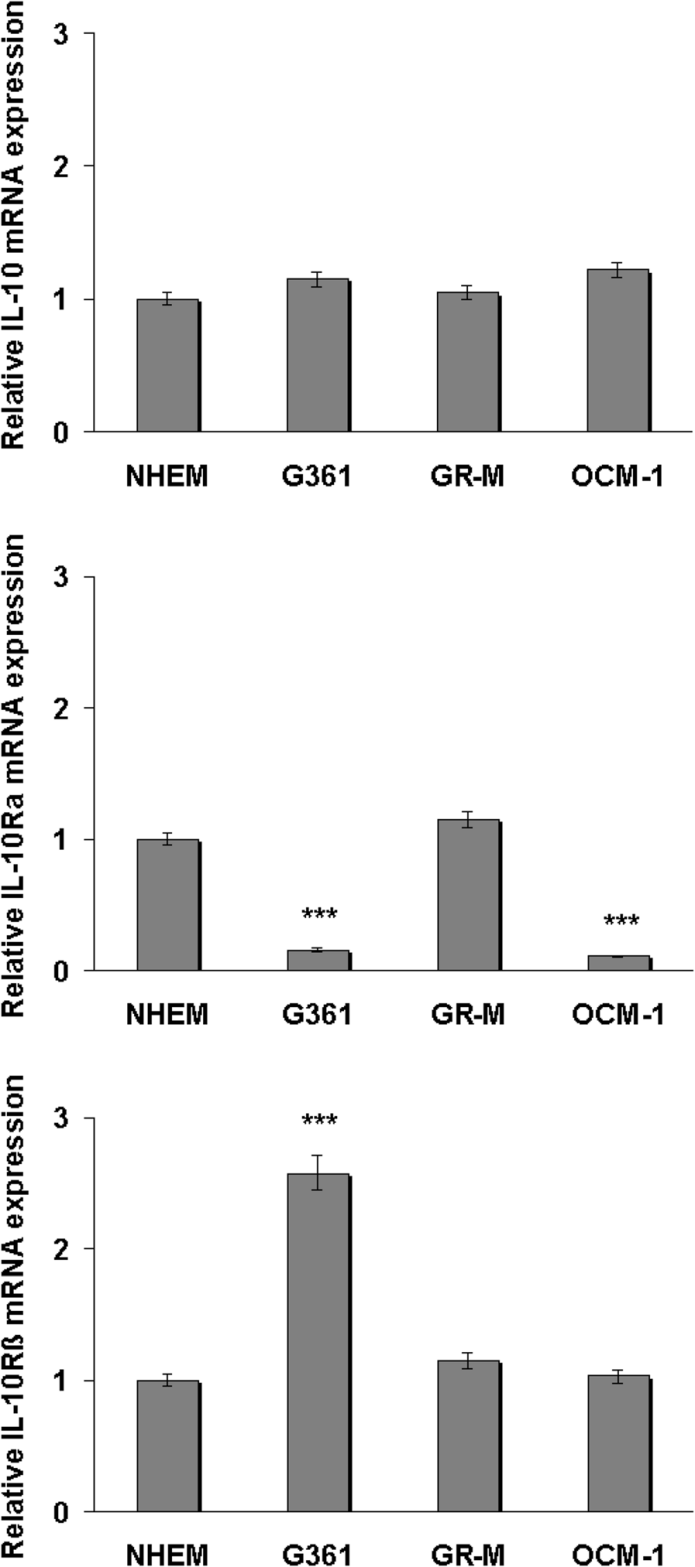
Expression levels of *IL-10*, *IL-10Rα*, and *IL-10Rβ* in cutaneous and uveal melanoma cell lines. Total RNA was extracted from cutaneous (G361 and GR-M) and uveal (OCM-1) melanoma cells, reverse-transcribed, and analyzed by qPCR. mRNA levels of *IL-10*, *IL-10Rα*, and *IL-10Rβ* were normalized by using the housekeeping gene β-actin as the inner control. Data are depicted as the mean ± SD of three independent experiments. Significant ***p < 0.001, as compared to NHEM cells

The mRNA content of *IL-10* and its receptor subunits *(IL-10Rα* and *IL-10Rβ*) was analyzed by quantitative PCR (qPCR) in two cutaneous (G361 and GR-M) and one uveal (OCM-1) melanoma cell lines and compared to that of NHEM. As shown in Fig. 1, levels of *IL-*10 mRNA expression did not significantly differ from that of NHEM in any of the cell lines examined. Lower amounts of *IL-10Rα* expression were found in G361 and OCM-1 cells, and higher levels of IL-10Rβ were observed in G361 cells. GR-M did not exhibit any modifications in *IL-10Rα* or *IL-10Rβ* expression.

### Prediction of miRNAs targeting *IL-10Rα* and *IL-10Rβ*

**Fig. 2:**
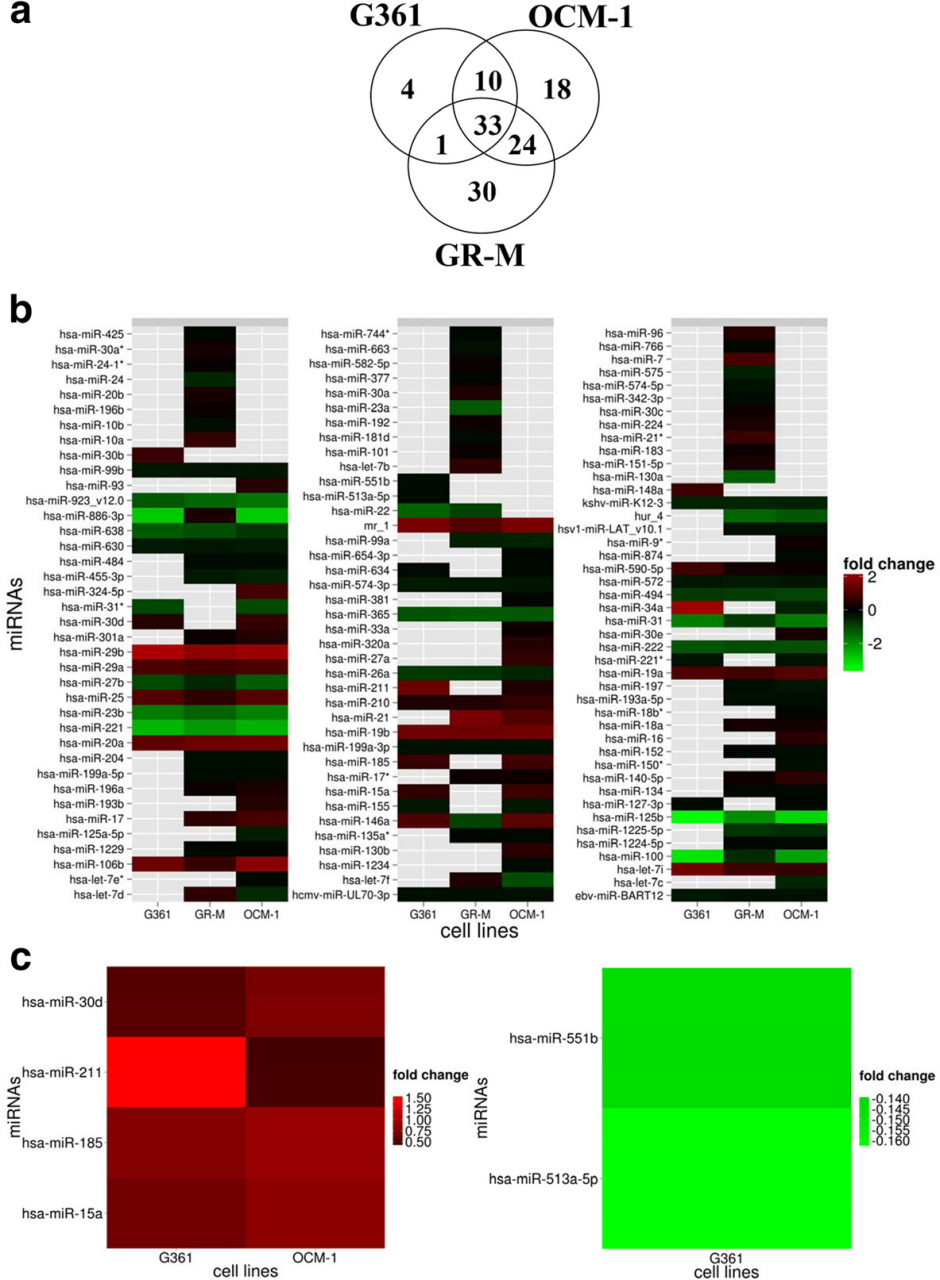
miRNA expression profiles in cutaneous and uveal melanoma. **a** Venn diagram illustrating the common and specific miRNAs in the indicated cells. **b** Heat map comparing the average fold-changes in microRNAs with significantly higher (*red*) or lower (*green*) expression in melanoma cells in comparison to normal human epidermal melanocytes (NHEM) (FDR ≤0.001). **c** Heat map relative to the 4 common miRNAs with up-regulated expression pattern in OCM-1 and G361 cells and the 2 common miRNAs down-regulated exclusively in OCM-1. FDR, false discovery rate

To test for possible post-transcriptional modulation of *IL-10Rα* and *IL-10Rβ* expression by miRNAs, genome-wide miRNA expression profiling was carried out. Figure 2 (sections B and C) shows that, as compared to NHEM, only 4 miRNAs (miR-15a, miR-185, miR-211, and miR-30d) were upregulated in G361 and OCM-1 cells, while remaining at similar levels in GR-M cells. Two miRNAs (miR-513a-5p and miR-551b) were down-regulated exclusively in G361 cells. The expression levels of these miRNAs were confirmed by qPCR (*data not shown*).

To predict miRNAs/mRNAs interactions, TargetScan (http://www.targetscan.org/), microRNA (www.microrna.org/) and PITA (http://genie.weizmann.ac.il/pubs/mir07/mir07_data.html) were used. Three out of the four miRNAs upregulated in G361 and OCM-1 and unchanged in GR-M were predicted to have seed regions able to bind to the 3′UTR of *IL-10Rα* (miR-15a was reported in all the miRNA target prediction systems, miR-185 in microRNA and PITA; miR-211 in microRNA and PITA). None of the miRNAs exclusively downregulated in G361 cells had *IL-10Rβ* as a putative target transcript.

### *IL-10Rα* is a target of miR15a, miR185, and miR211

**Fig. 3:**
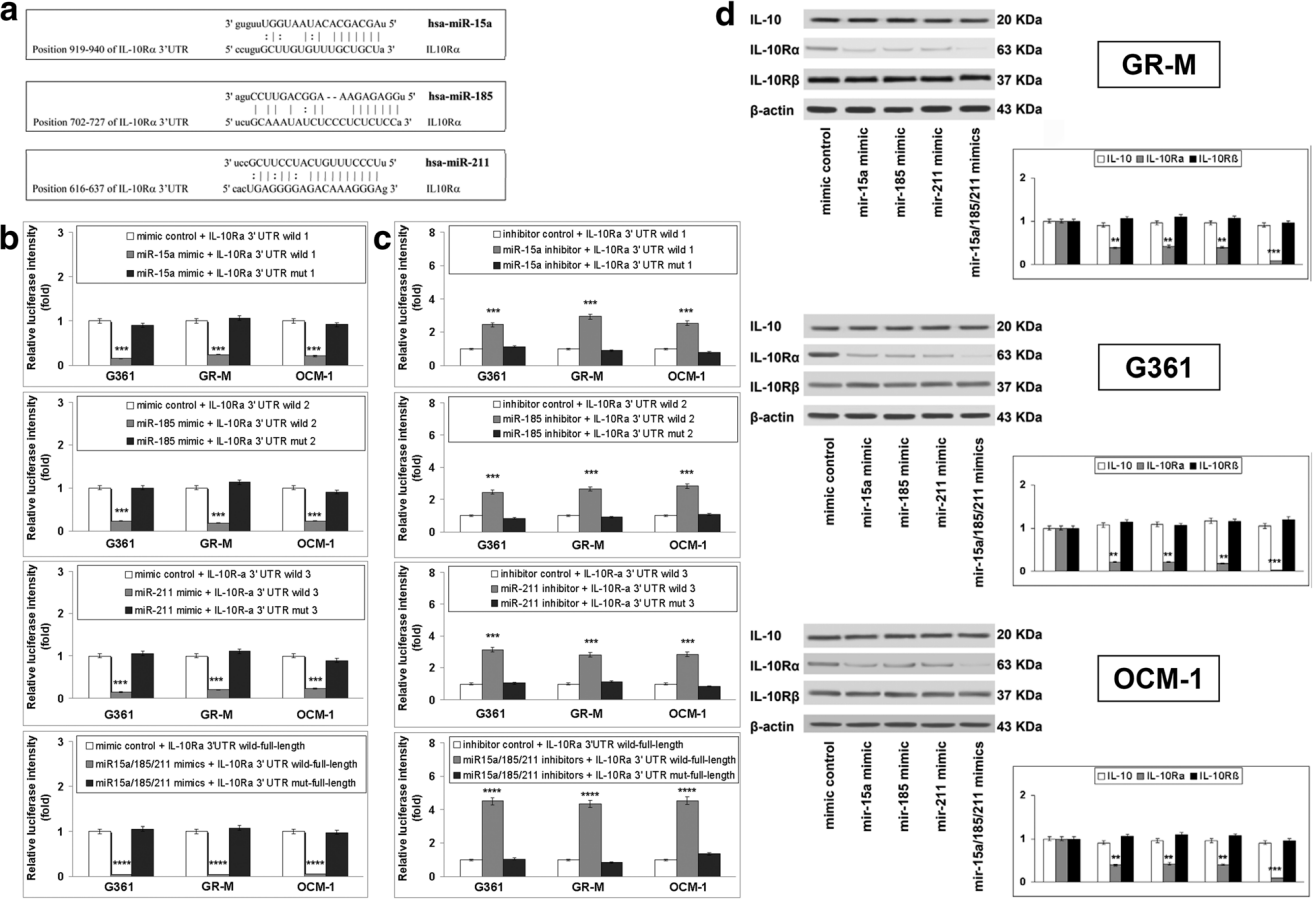
*IL-10Rα* is the direct target of miR-15a, miR-185, and miR-211 **a** Schematic representation of the predicted interaction of miR-15a, miR-185, and miR-211 with *IL-10Rα* 3′UTR site. **b** Luciferase reporter assay was performed to detect the effect of individual or combined miRNA mimics on the luciferase intensity controlled by 3′UTR of *IL-10Rα.* Data are depicted as the mean ± SD of three independent experiments. Significant ***p < 0.001 and ****p < 0.0001, as compared to cells transfected with mimic control. **c** Luciferase reporter assay was performed to detect the effect of individual or combined miRNA inhibitors on the luciferase intensity controlled by 3′UTR of *IL-10Rα*. Data are depicted as the mean ± SD of three independent experiments. Significant ***p < 0.001 and ****p < 0.0001, as compared to cells transfected with inhibitor control. **d** Cells were transfected with individual or combined miRNA mimics and subjected to western blot for IL-10, IL-10Rα, and IL-10Rβ protein expression. β-actin was used as an internal control for normalization. Blots are representative of three independent experiments. The relative densities were calculated by dividing the density of IL-10Rα bands by the density of β-actin band at the same point. Significant **p < 0.01 and *** *p* < 0.001, as compared to cells transfected with mimic control

To validate the direct interaction of miR15a, miR185, and miR211 with *IL-10Rα* mRNA (Fig. 3a), we constructed a luciferase reporter system containing a binding site (IL-10Rα-3′-UTR-wt) or a mutated site (IL-10Rα-3′-UTR-mut). The vectors were co-transfected into G361, GR-M, and OCM-1 cells with miR15a, miR185, and miR211 mimics or inhibitors. The luciferase activity of IL-10Rα-3′-UTR-wt in cells transfected with miR-15a, or miR-185, or miR-211 mimics was significantly decreased (*p* < 0.001), compared to negative controls. When the three mimics were co-transfected, luciferase activity was almost abolished. However, none of them, either alone or in combination, affected the transcription of the pGL3-IL-10Rα-mut vector (Fig. 3b). When blocking the expression of the three miRNAs with specific inhibitors, luciferase intensity markedly increased in cells transfected with the IL-10Rα-3′-UTR-wt vector (*p* < 0.001). This increase was higher when cells were co-transfected with all the three inhibitors. No change in cells transfected with pGL3-IL-10Rα-mut (Fig. 3c) was observed. These results suggest that the 3′-UTR of *IL-10Rα* mRNA might be the target of miR15a, miR185, and miR211. Next, we further investigated the regulation of *IL-10Rα* protein expression by miR-15a, miR-185, and miR-211. Western blot showed that the IL- 10Rα expression significantly decreased in cells transfected with individual mimics and was almost abolished by their combination. IL-10 and IL-10Rβ were not affected by any of the miRNA mimics either alone or in combination (Fig. 3d).

### The IL-10/IL-10R system and miR-15a, miR-185, miR-211 expression in cutaneous and uveal melanoma samples

**Fig. 4:**
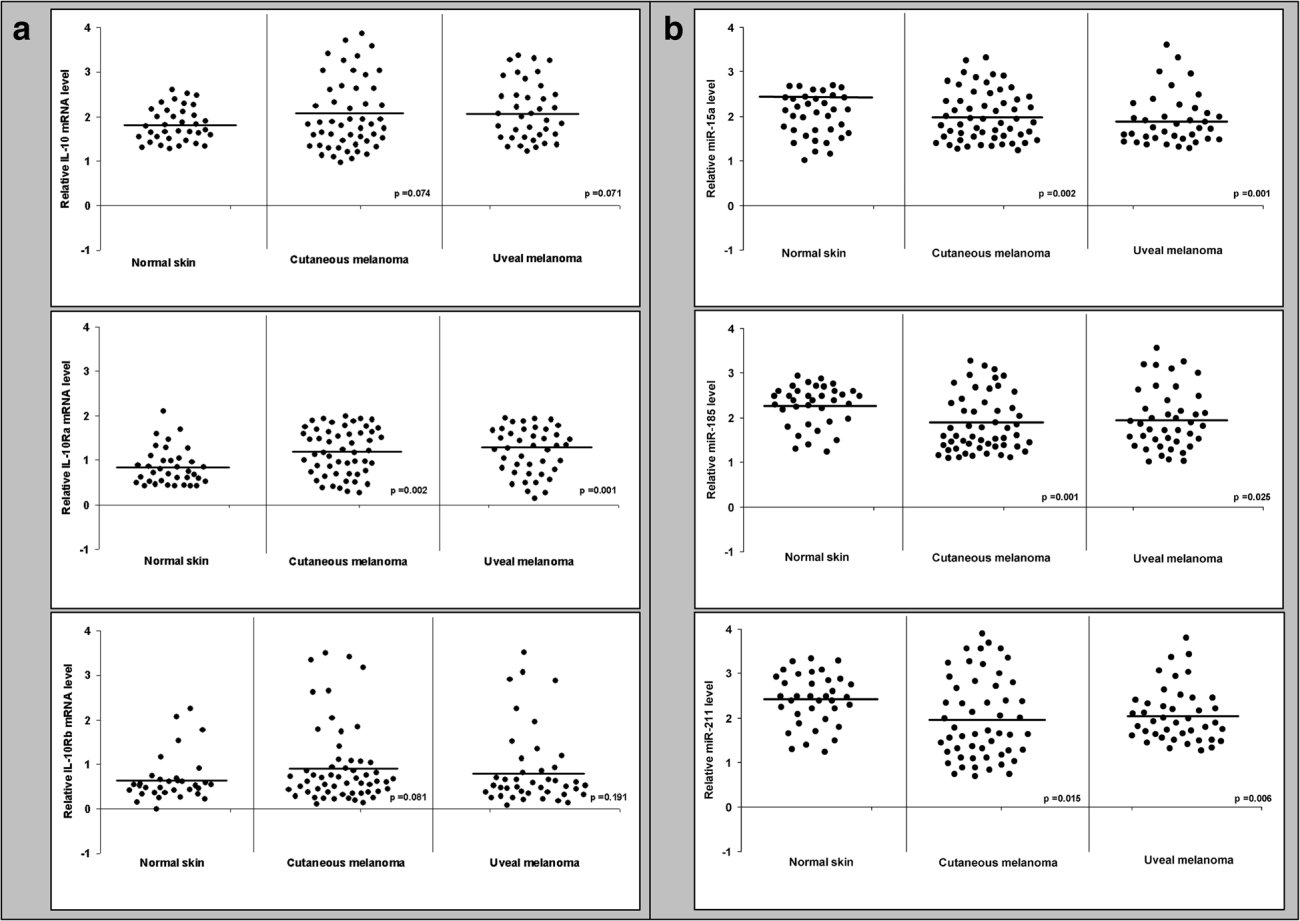
IL-10/IL-10R system and miR-15a, miR-185, miR-211 expression in cutaneous and uveal melanoma samples. Total RNA was extracted from 35 normal skin specimens, 52 cutaneous melanomas, and 41 uveal melanomas, reverse-transcribed, and analyzed by qPCR to determine the relative amounts of IL-10, IL-10Rα, IL-10Rβ mRNA (**a**) and miR-15a, miR-185, miR-211 (**b**). Data are representative of three independent experiments. Means of the measurements are shown with black lines

Next, we proceeded to explore the expression of the members of IL-10/IL-10R system, miR-15a, miR-185, and miR-211 in cutaneous and uveal melanoma samples as compared to normal skin. Figure 4 shows significant higher levels of IL-10Rα (section A) accompanied by a correspondent decrease in miR-15a, miR-185, and miR-211 (section B) in tumor specimens. IL-10 and IL-10Rβ did not show significant modification in their expression levels (section A).

### Effects of miR-15a, miR-185, and miR-211 on IL-10-induced melanoma cell proliferation

**Fig. 5:**
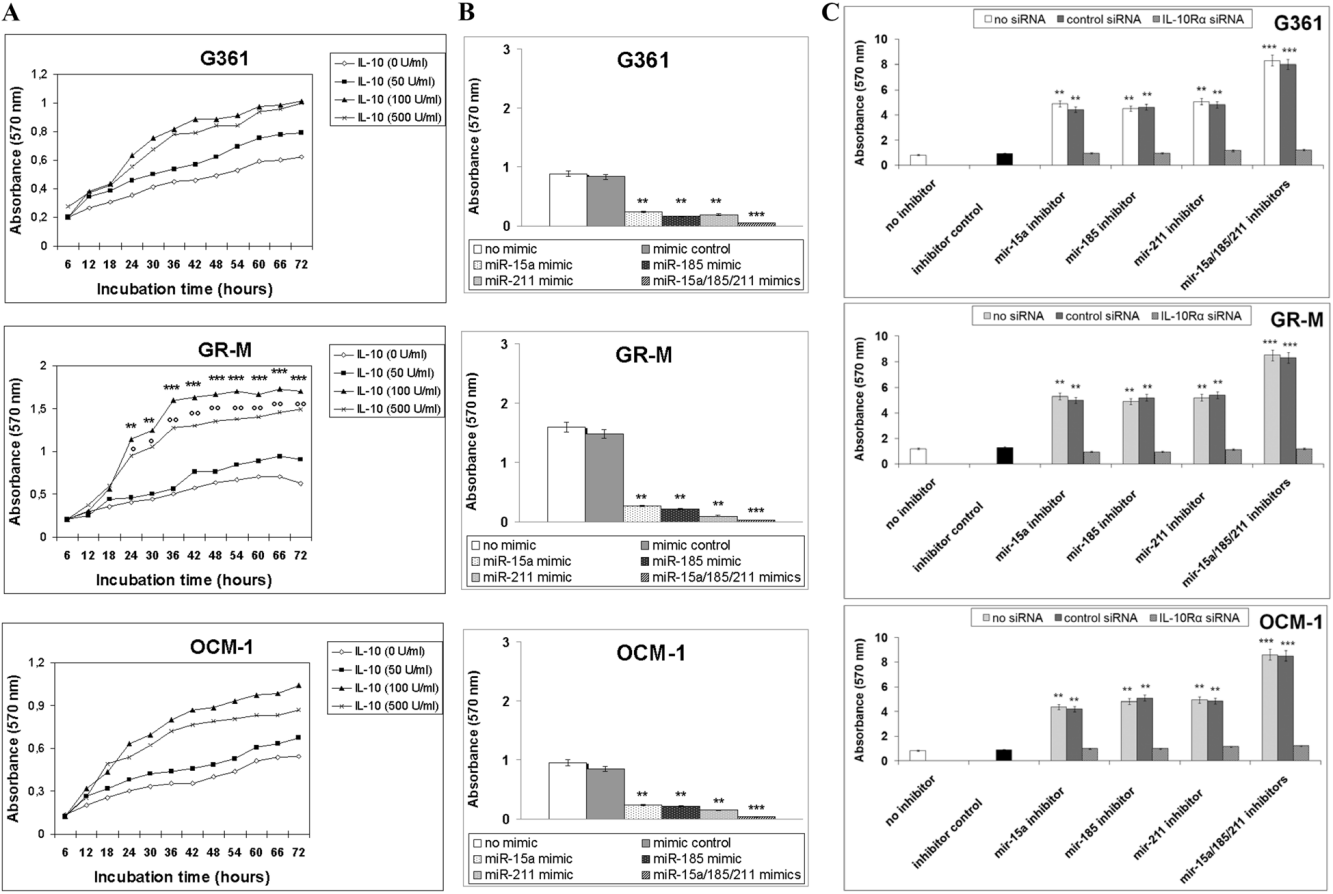
Effects of miR-15a, miR-185, and miR-211 on IL-10-induced melanoma cell growth. **a** Cells were cultured in the presence of various doses of rIL-10: 0 (as negative control), 50, 100, or 500 U/ml and proliferation was assessed by MTT assay. Absorbance at 570 nm was measured at 6-hr intervals over a 72-hr culture period. Each experiment was repeated three times, and data represent the mean ± SD of three determinations. Significant **p < 0.01 and ***p < 0.001, cells treated with 100 U/ml as compared to the negative control at the same time point. Significant °*p* < 0.05 and °°*p* < 0.01, cells treated with 500 U/ml as compared to the negative control at the same time point. **b** Cells were transfected with individual or combined miR-15a, miR-185, and miR-211 mimics for 48-hr and then treated with rIL-10 (100 U/ml) for 36-hr. Proliferation was determined as reported in section A. Significant **p < 0.01 and ***p < 0.001, as compared to untransfected cells. **c** Cells were transfected with individual or combined miR-15a, miR-185, and miR-211 inhibitors for 48-hr and, where indicated, co-transfected with siRNA against IL-10Rα or non-targeting control siRNA. Then, cells were treated with rIL-10 (100 U/ml) for 36-hr. Proliferation was determined as reported in section A. Significant **p < 0.01 and ***p < 0.001, as compared to untransfected cells

As the melanoma environment is characterized by high levels of IL-10 [14], we analyzed the effects of miR-15a, miR-185, and miR-211 on the growth of melanoma cells exposed to this cytokine. Firstly, cells were cultured in presence of various doses of IL-10 (50, 100, or 500 U/ml), and the proliferation was determined at 6-hr intervals during a 72-hr culture period. As seen in Fig. 5a, relative to cells with medium alone, the proliferation of GR-M cells dramatically increased during the 12–48 h interval following treatment with IL-10 at concentrations ranging from100 through 500 U/ml. G361 and OCM-1 cells showed a slight, non-significant increase in cell growth after exposure to IL-10. These data show that IL-10 was able to induce optimal proliferative effects in GR-M cells already at a concentration of 100 U/ml in agreement with previous studies using T cells [15] and that this increase was also observed at 500 U/ml. Therefore, treatment with100 U/ml of IL-10 for 36-hr was used for cell proliferation assays in the presence of miR-15a, miR-185, and miR-211 mimics or inhibitors. Proliferation was reduced after transfection of all the cell lines with individual miRNA mimics and almost abolished following co-transfection with the combined mimics (Fig. 5b). Knockdown of miR-15a, or miR-185, or miR-211 markedly promoted the proliferation in all the cell lines and the combined inhibitors further increased growth. The proliferative effects of miRNA inhibitors, either alone or in combination, were abolished when IL-10Rα was silenced by the specific siRNA (Fig. 5c).

## Discussion

IL-10 has been extensively studied for its ability to affect immune responses against a wide variety of cancers, and, to a lesser extent, also against the melanoma [4]. Regarding this tumor, conflicting findings are reported, some of which showing that IL-10 significantly contributes to melanoma development [7, 9], and some others indicating that this cytokine strengthens the effects of anti-melanoma vaccines and NK cells [6, 10]. Moreover, what are the direct effects of IL-10 on melanoma cells and whether the latter differ from normal melanocytes in their responsiveness to the cytokine is presently unclear. Here we did not observe any significant changes in *IL-10* expression in the melanoma cell lines studied relative to normal melanocytes (Fig. 1). However, literature data are contradictory in this regard, as some papers report that IL-10 is an autocrine melanoma growth factor [7, 16], others obtained uneven results in their melanoma cell systems [5], and still others found that IL-10, as well as its receptor, are expressed at lower levels in melanoma samples than in healthy skin [14]. In this context, a more prominent role seemed to be played by *IL-10Rα* expression than by IL-10 production, according to our data (Fig. 1). Indeed, the uveal and cutaneous melanoma cell lines OCM-1 and G361, which displayed downregulation of *IL-10Rα* (with G361 also exhibiting higher *IL-10Rβ* expression), did not proliferate after IL-10 treatment, as opposed to GR-M cells with moderate *IL-10Rα* expression (Fig. 5). The observation that the two cutaneous melanoma cell lines exhibit a different expression profile and that the cutaneous melanoma line G361 is similar to the uveal melanoma cell line OCM-1 with respect to *IL-10Rα* and miRNA expression and responses to IL-10 is not surprising. Indeed, G361 showed a different behavior from GR-M in endogenous retrovirus K protein Rec expression upon exposure to UV [17] and from several other cutaneous melanoma cell lines in the expression of ATP-binding cassette (ABC) B5 mRNA [18]. On the other hand, G361 exhibited an expression pattern of *DcR1* and *DcR2* identical to that of uveal melanoma cell lines as a result of promoter hypermethylation and a likewise identical responsiveness to the demethylating agent 5-aza-dC [19]. IL-10R is expressed on a variety of cells, in particular immune cells, including most hematopoietic cells such as monocytes, macrophages, and T- and B-lymphocytes [4], but is also expressed on non-hematopoietic cells, such as epidermal cells or keratinocytes [5]. Only two studies evaluated the expression of IL-10R on melanoma cells [7, 14]. In an early study, a comparison was not carried out with normal melanocytes and no distinction was made between the two IL-10R chains [7]. A second, more recent study*,* after comparing neoplastic melanocytes with normal skin, reported a great variability in IL-10R expression, with lower levels of in melanoma, but, again, without a distinction between α and β chains [14]. This is a critical gap in the understanding of the role of the IL-10/IL-10R system in melanoma, since α and β chains are encoded by distinct genes and display different role in triggering the signaling events [4]. In this regard, here we showed that the expression of *IL-10Rα* is crucial for the melanoma cell responsiveness to IL-10.

Melanocytes play a central role in skin innate immune system and inflammation [20] to the point that they have been defined “a sensitive barometer of inflammatory conditions of the skin” [21]. The recently evidenced role of miRNA dysregulation in melanoma [11, 22], prompted us to investigate whether differential expression of the two chains of IL-10R occurring in the three different melanoma models was in association with specific miRNA profiles. We found that as many as three out of four miRNAs (namely miR-15a, miR-185, and miR-211) were increased in cells with low expression of IL-10Rα, and that these regulatory molecules targeted the *IL-10Rα* gene (Fig. 2). This conclusion stemmed from a series of experiments based on miRNA target prediction tools, miRNA target reporter assay, and western blot (Fig. 3). The amounts of IL-10 and IL-10Rβ proteins were not affected by the three miRNAs. The expression profile of IL-10/IL-10R, miR-15a, miR-185, and miR-211 observed in cutaneous and uveal melanoma tissues exhibited the same trend observed in melanoma cell lines (Fig. 4). Interestingly, miR-15a, miR-185, and miR-211 are thought to function as tumor suppressors in melanocytes, as they repress many genes implicated in the melanomagenesis. For example, downregulation of miR-15a, which is observed in several cancers including melanoma, led to an overexpression of several oncogens, such as *Bcl-2*, *Cyclin D1*, and *Mcl-1* [23]. In addition, miR-185 exhibits strong anti-proliferative effects on melanoma either *in-vitro* or *in vivo* [24] and miR-211 inhibits the aggressive and invasive phenotype of melanoma cells [11]. However, to our knowledge, the regulation of *IL-10Rα* expression by these three miRNAs and the possible significance of *IL-10Rα* downregulation in melanoma cell proliferation have not yet been addressed. Here we showed that miR-15a, miR-185, and miR-211 mimics inhibited and miRNA inhibitors increased the proliferation of IL-10-treated melanoma cells through IL-10 signaling, since the silencing of *IL-10Rα* cancelled the effects of miRNA inhibitors on the proliferation (Fig. 5). Another interesting finding from these experiments is that the three miRNAs considered exerted synergistic effects both in modulating the expression of IL-10Rα or in regulating the proliferation of melanoma cells. These data suggest the potential usefulness of a combined therapeutic strategy targeted to the expression of miR-15a, miR-185, and miR-211 in melanoma cells.

Data presented here acquire perhaps further relevance in light of a recent paper showing that the expression of IL-10 and IL-10R, despite the great variability, is lower in melanomas compared to normal skin [14]. In this context, our findings give emphasis to whether or not melanoma cells express *IL-10Rα*, as the expressing cells proliferated in the presence of IL-10 – which may be produced by melanoma environment – while the non-expressing melanoma cells were unresponsive to the cytokine. Therefore, our data add to our knowledge about the significance of *IL-10Rα* post-transcriptional regulation in the characteristics of malignancy of melanoma, independently from the questioned immunostimulant or immunosuppressive role of IL-10.

## Conclusions

Taken together, findings reported in the present study suggest that the *IL-10Rα* expression in melanoma cells is post-transcriptionally regulated by miR-15a, miR-185, and miR-211. The variability in IL-10Rα mRNA amounts we observed is correlated with the differences in cell growth in response to IL-10. Thus, it could be argued the IL-10/IL-10 receptor system may become a new epigenetic therapeutic target for melanoma treatment.

## Availability of supporting data

All microarray data of this paper have been deposited in the LabArchives, LLC (http://www.labarchives.com/), under doi :10.6070/H4C53HWK.

## Abbreviations

Interleukin10: IL-10
MTT: 3-(4,5-dimethylthiazol-2-yl)-2,5-diphenyltetrazolium bromide
IL-10 receptor: IL-10R
miRNAs: microRNAs
qPCR: Quantitative PCR
Bcl-2: B-cell CLL/lymphoma 2
Mcl-1: Myeloid cell leukemia 1
